# Communication technology for eye care

**Published:** 2022-06-07

**Authors:** Kriti Shukla, Yuddha Dhoj Sapkota, Anthony Vipin Das, Priya Morjaria

**Affiliations:** 1 Indian Institute of Public Health.; 2 IAPB South East Asia.; 3 LV Prasad Eye Institute.; 4 London School of Hygiene & Tropical Medicine and Peek Vision.

**Communication technology has great potential to improve access to eye health care, provided equity of access is a priority.**

**Figure:**
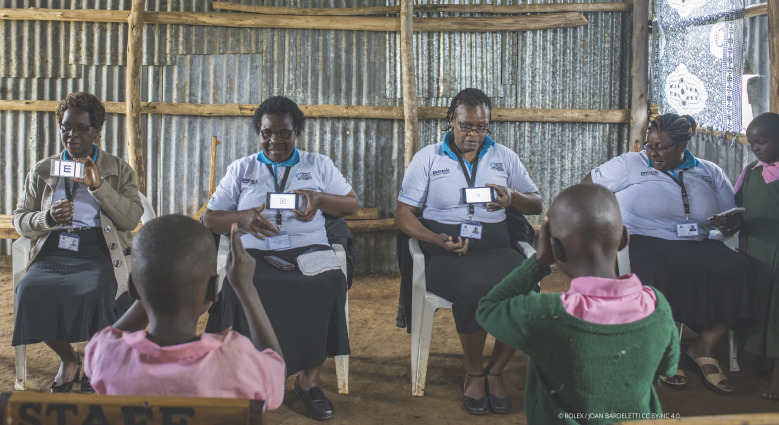
Communication technologies such as smartphone apps help to connect people to the eye care they need. **KENYA**

Digital technologies are part of our life, and they have tremendous potential to improve people's health if applied in the health sector. The Global Strategy on Digital Health (see bit.ly/digi-WHO), adopted in 2020 by the World Health Assembly, supports the strengthening of digital health services to improve health outcomes. There is also growing consensus that using cutting-edge digital innovations and technologies will enable more people to benefit from universal health coverage.

Digital health is an umbrella term that includes communication technology, health information technology, big data, artificial intelligence, genomics, and wearable technology. In this issue, our focus is specifically on communication technologies such as mobile health (mHealth), telehealth, telemedicine, and teleconsultations. These have become vital tools for delivering health care services, in part due to the pressures brought by the COVID-19 pandemic.

Communication technology has great potential to help deliver good quality and affordable health care. However, there are challenges. New technology can be expensive and must therefore be well suited to the needs of the community where it will be used, and of sufficient quality to justify the financial investment made, as our article on refractive error innovations demonstrates. Another major challenge is pre-existing inequalities in communities’ access to education, infrastructure, and technology. Appropriate, equitable, and ethical use of technology is a must if we are to avoid deepening already existing health inequities. Factors such poor internet connectivity, low digital literacy, and lack of access to broadband internet and smartphones – known as the ‘digital determinants of health’ – should be central in our thinking when incorporating communication technology into existing services.

In this issue, you will therefore find articles that provide guidelines for developing inclusive and accessible teleophthalmology services for people with disabilities, those with low digital literacy, and those who lack internet access, while protecting patients’ data and privacy. We also discuss artificial intelligence (AI) in eye care and the need for equitable development of AI services.

We hope that you will find useful ideas and inspiration in articles from different regions that show the potential of AI, mHealth and teleconsultations to bring patients closer to the eye care they need.

**Figure:**
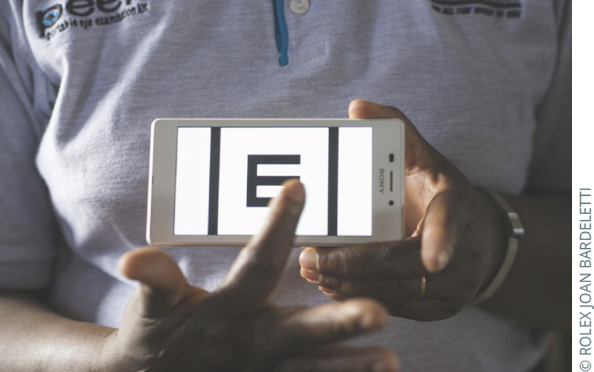
Woman holding a smartphone showing the letter ‘E’ and swiping the screen. **KENYA**

